# Intracranial Hypertension in Children without Papilledema

**DOI:** 10.15844/pedneurbriefs-29-8-4

**Published:** 2015-08

**Authors:** Ana B. Chelse, Leon G. Epstein

**Affiliations:** 1 Division of Neurology, Ann & Robert H. Lurie Children's Hospital of Chicago, Chicago, IL; 2 Departments of Pediatrics and Neurology, Northwestern University Feinberg School of Medicine, Chicago, IL

**Keywords:** Headache, Intracranial Hypertension, Pediatric, Pseudotumor Cerebri

## Abstract

Researchers at Nationwide Children's Hospital studied the frequency of intracranial hypertension without papilledema in children followed in a multispecialty pediatric intracranial hypertension clinic.

Researchers at Nationwide Children's Hospital studied the frequency of intracranial hypertension without papilledema in children followed in a multispecialty pediatric intracranial hypertension clinic. Children aged 2 to 22 years (mean age of 12 years) were diagnosed with intracranial hypertension by 2 neurologists based on history, physical exam and opening pressure measurements on lumbar puncture. An eye exam by an ophthalmologist determined the presence or absence of papilledema. The study included both primary and secondary intracranial hypertension. Patients were divided into 2 groups depending on the presence or absence of papilledema. The primary outcome was to determine the frequency of intracranial hypertension without papilledema in children. 152 patients were included in the study. Group 1 consisted of 27 (17.8%) patients who met the criteria of intracranial hypertension without papilledema. Group 1 was compared to 125 (82%) children with intracranial hypertension and the presence of papilledema. Groups did not differ significantly in age, opening pressure, or body mass index. [1]

COMMENTARY. Aylward and colleagues concluded that 17.8% of children with intracranial hypertension did not have papilledema. These results are in line with previously published rates in pediatric and adult studies. Previous studies and case reports have reported rates of intracranial hypertension without papilledema ranges from 5.7% to 48% [2–5]. A large review of 353 adult and pediatric patients with idiopathic intracranial hypertension at the University of Utah showed a frequency of 5.7% (20/353) of patients with intracranial hypertension lacked papilledema [2]. The Utah study reported a lower opening pressure (30.9 cm of water vs 37.3 cm) in patients without papilledema [2]. Among 27 pediatric patients with intracranial hypertension in a small University of Texas-Houston study, 48% (13/27) of patients who met the diagnostic criteria for intracranial hypertension lacked papilledema [5]. The current study had missing variables for BMI and initial opening pressure in 15.8% and 9.3% of patients, respectfully. Inclusion of these variables may have changed the author's reported frequency rate. Previous research reported normal ranges of OP in both sedated and nonsedated patients. Avery et al included sedated and nonsedated study patients and determined the upper limits of normal for OPs in children to be 28 cm of water without anesthesia and 30 cm with anesthesia [6]. Other papers cited in the current study used lower OP values as the upper limit of normal. As lower ranges of normal OP were used, the percentage of pediatric patients with increased pressure in the absence of papilledema was likely overstated. Current research suggests that pediatric lumbar puncture OPs are not significantly lower than those of adults and are not strongly related with age or body mass index [6, 7]. Taking the present data and previous reports into consideration, physicians should not dismiss the possibility of intracranial hypertension in patients with symptoms suggesting intracranial hypertension based solely on the presence or absence of papilledema. Intracranial hypertension should be considered in children with persistent headaches refractory to standard management.
